# Evolutionary and Genomic Insights into *Clostridioides difficile* Sequence Type 11: a Diverse Zoonotic and Antimicrobial-Resistant Lineage of Global One Health Importance

**DOI:** 10.1128/mBio.00446-19

**Published:** 2019-04-16

**Authors:** Daniel R. Knight, Brian Kullin, Grace O. Androga, Frederic Barbut, Catherine Eckert, Stuart Johnson, Patrizia Spigaglia, Kazuhiro Tateda, Pei-Jane Tsai, Thomas V. Riley

**Affiliations:** a Medical, Molecular and Forensic Sciences, Murdoch University, Western Australia, Australia; b Department of Molecular and Cell Biology, University of Cape Town, Cape Town, South Africa; c School of Biomedical Sciences, The University of Western Australia, Nedlands, Western Australia, Australia; d Diagnostic Genomics, PathWest Laboratory Medicine, Queen Elizabeth II Medical Centre, Nedlands, Australia; e National Reference Laboratory for *C. difficile*, Hospital Saint-Antoine, Paris, France; f Sorbonne Université, Centre d'Immunologie et des Maladies Infectieuses-Paris, Département de Bactériologie, Hôpitaux Universitaires de l’Est Parisien, Paris, France; g Loyola University Medical Center and Hines Veteran Affairs Hospital, Hines, Illinois, USA; h Department of Infectious Diseases, Istituto Superiore di Sanità, Rome, Italy; i Department of Microbiology and Infectious Diseases, Toho University School of Medicine, Tokyo, Japan; j Department of Microbiology and Immunology, National Cheng Kung University, Medical College, Tainan, Taiwan; k School of Medical and Health Sciences, Edith Cowan University, Joondalup, Western Australia, Australia; l Department of Microbiology, PathWest Laboratory Medicine, Queen Elizabeth II Medical Centre, Nedlands, Western Australia, Australia; Brigham and Women's Hospital; Mayo Clinic; Leiden University Medical Center

**Keywords:** antimicrobial resistance, *Clostridium difficile*, epidemiology, evolution, livestock, microbial genomics, One Health, toxin, transmission, zoonosis

## Abstract

Clostridioides difficile (Clostridium difficile) sequence type 11 (ST11) is well established in production animal populations worldwide and contributes considerably to the global burden of C. difficile infection (CDI) in humans. Increasing evidence of shared ancestry and genetic overlap of PCR ribotype 078 (RT078), the most common ST11 sublineage, between human and animal populations suggests that CDI may be a zoonosis. We performed whole-genome sequencing (WGS) on a collection of 207 ST11 and closely related ST258 isolates of human and veterinary/environmental origin, comprising 16 RTs collected from Australia, Asia, Europe, and North America. Core genome single nucleotide variant (SNV) analysis identified multiple intraspecies and interspecies clonal groups (isolates separated by ≤2 core genome SNVs) in all the major RT sublineages: 078, 126, 127, 033, and 288. Clonal groups comprised isolates spread across different states, countries, and continents, indicative of reciprocal long-range dissemination and possible zoonotic/anthroponotic transmission. Antimicrobial resistance genotypes and phenotypes varied across host species, geographic regions, and RTs and included macrolide/lincosamide resistance (Tn*6194* [*ermB*]), tetracycline resistance (Tn*6190* [*tetM*] and Tn*6164* [*tet44*]), and fluoroquinolone resistance (*gyrA*/*B* mutations), as well as numerous aminoglycoside resistance cassettes. The population was defined by a large “open” pan-genome (10,378 genes), a remarkably small core genome of 2,058 genes (only 19.8% of the gene pool), and an accessory genome containing a large and diverse collection of important prophages of the *Siphoviridae* and *Myoviridae*. This study provides novel insights into strain relatedness and genetic variability of C. difficile ST11, a lineage of global One Health importance.

## Abstract (precis)

Historically, *Clostridioides difficile* (*Clostridium difficile*) has been associated with life-threatening diarrhea in hospitalized patients. Increasing rates of C. difficile infection (CDI) in the community suggest exposure to C. difficile reservoirs outside the hospital, including animals, the environment, or food. C. difficile sequence type 11 (ST11) is known to infect/colonize livestock worldwide and comprises multiple ribotypes, many of which cause disease in humans, suggesting CDI may be a zoonosis. Using high-resolution genomics, we investigated the evolution and zoonotic potential of ST11 and a new closely related ST258 lineage sourced from diverse origins. We found multiple intra- and interspecies clonal transmission events in all ribotype sublineages. Clones were spread across multiple continents, often without any health care association, indicative of zoonotic/anthroponotic long-range dissemination in the community. ST11 possesses a massive pan-genome and numerous clinically important antimicrobial resistance elements and prophages, which likely contribute to the success of this globally disseminated lineage of One Health importance.

## INTRODUCTION

*Clostridioides difficile* (*Clostridium difficile*) is a toxin-producing antimicrobial-resistant (AMR) enteropathogen historically associated with diarrhea and pseudomembranous colitis in hospitalized patients (1). In both the northern hemisphere and Australia, community-acquired C. difficile infection (CA-CDI) currently accounts for around a third of all CDI cases, and production animals have been identified as potential sources/reservoirs (1, 2). As shown by both conventional PCR and fine-scale whole-genome sequencing (WGS) approaches, the recovery of indistinguishable C. difficile strains from human and livestock populations indicates that CDI may be a zoonosis (2–5).

C. difficile multilocus sequence type 11 (MLST 11 [here ST11]) is a diverse evolutionary lineage of global One Health importance (3, 6, 7). The principal ST11 strain lineage, PCR ribotype 078 (RT078), is well established in porcine and bovine populations throughout North America and Europe and is responsible for much of the human CA-CDI in these regions (2, 6, 8, 9). Indeed, RT078 is now the third most frequently isolated RT in human CDI in Europe (8, 10). RT078 has never been detected in Australian livestock and causes only sporadic cases of CDI in human populations in Australia. However, neonatal calves and piglets in Australia are reservoirs for a number of other ST11 RTs, notably 126, 127, 033, and 288 (11–13), all of which have been isolated from humans with CDI in Australia, Europe, the Middle East, and Asia (1, 2). While there has been a focus on the genome and evolution of RT078 (4, 14–16), no such studies have been performed on other ST11 RT sublineages.

**FIG 1:**
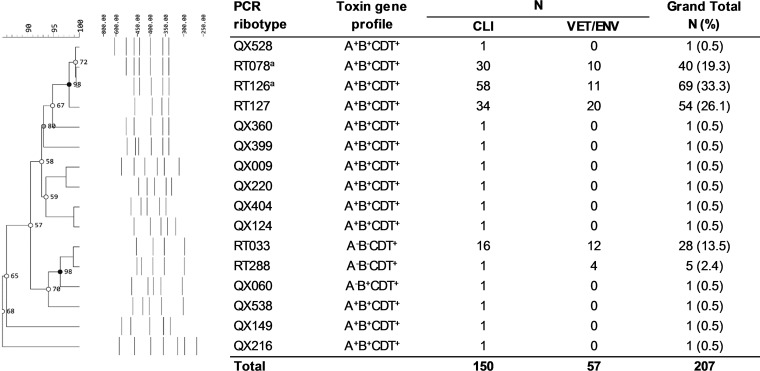
Molecular epidemiology. PCR ribotyping banding patterns for 16 unique C. difficile RTs analyzed in this study (*n = *207). The corresponding toxin gene profile is also provided. Origin: CLIN, clinical; VET, veterinary; ENV, environmental; RT, PCR ribotype; QX, novel RT assignment (internal nomenclature). Superscript a indicates the molecular epidemiology for some of these strains is based on previously published works (4, 36).

To address this knowledge gap, we used high-resolution genomics to investigate the genetic diversity, evolution, and zoonotic potential of a collection of 207 C. difficile ST11 strains of human clinical, veterinary, and environmental origins. These strains originated from Australia, Asia, Europe, and North America, were collected between 1980 and 2016, and comprised 16 RTs, including major ST11 sublineages 078, 126, 127, 033, and 288 (*n = *196) and 11 unique RTs not previously reported in our laboratory (Fig. 1). A detailed summary of strains and epidemiological data is provided in Data Set S1 hosted at figshare (https://doi.org/10.6084/m9.figshare.4822255) and outlined in Materials and Methods.

## RESULTS

Of the 207 sequenced genomes, 200 (96.6%) were confirmed *in silico* as ST11 and as belonging to evolutionary clade 5. The remainder (*n = *7 [3.4%], including six RT126 strains and one QX360 strain, all Australian in origin) were assigned to a novel clade 5 lineage, ST258 (see Data Set S1 at figshare [https://doi.org/10.6084/m9.figshare.4822255]). ST258 is a double-locus variant of ST11, differing in just 3 of 3,501 nucleotides (*atpA*, C138T and T205C; *tpi*, G326A) (0.09%). This finding was somewhat unexpected as, to date, all RT126 strains have been reported as ST11. For a global phylogenetic context, the relative evolutionary relatedness of STs 11 and 258, as well as other prominent STs from clades 1 to 4, is shown in Fig. S1 in Data Set S1. The RT was confirmed for these strains (see Fig. S2 in Data Set S1), and given the close evolutionary relationship to ST11, they were included in all subsequent analyses. WGS metrics, MLST data, and features for the 207 genomes evaluated in this study are summarized in Data Set S1.

### Intra- and interspecies transmission of globally disseminated *C. difficile* clade 5 clones.

**FIG 2:**
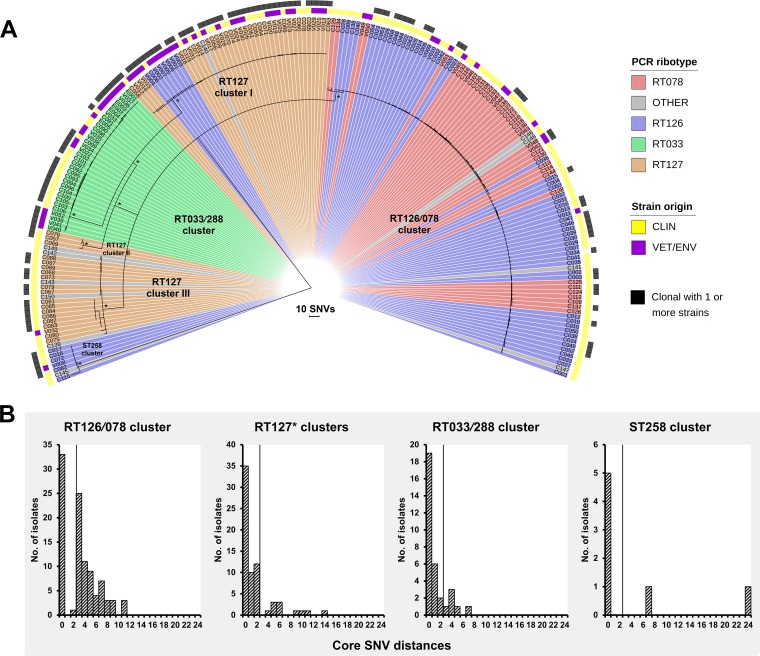
Microevolutionary analysis and clonal transmission. (A) Maximum likelihood phylogeny of 207 C. difficile genomes based on evolution in 1,076 nonrecombinant, nonrepetitive core genome SNVs in clonal frame. Taxa are colored according to RT lineage: RT033/288 (green; *n = *33), RT078 (red; *n = *40), RT126 (blue; *n = *69), RT127 (orange; *n = *54), or other (gray; *n = *11). Strain origin is indicated in yellow (clinical, taxa prefixed with “C”) and purple (veterinary/environmental, taxa prefixed with “V/E”). Clonal relationships (two or more strains sharing ≤2 core genome SNVs) are indicated in black. The tree is midpoint rooted, and the nodes are supported by 1,000 nonparametric bootstrap replicates (values of >95 are shown [*]). The overall topology supports PCR ribotype assignment with six major strain clusters identified (the RT126/078 cluster, RT127 clusters I to III, the RT033/288 cluster, and the sequence type 258 [ST258] cluster). (B) Distribution plots showing core genome SNV distances between each strain and the genetically closest strain in each cluster. Vertical lines represent the 2-SNV cutoff for the identification of clonally transmitted strains (18). In “RT127*,” the asterisk indicates that clusters I to III are merged.

The phylogenetic structure and clonal subpopulations of the 200 ST11 and 7 ST258 strains were explored by high-resolution core genome single nucleotide variant (SNV) analysis. WGS reads were mapped to the finished chromosome of C. difficile RT078 strain M120 (ST11 [NC_017174]) to a median depth of 100×. After filtering for indels, repetitive regions, mobile genetic elements, and putative recombination regions, a total of 1,076 high-quality SNVs in the clonal frame were found across the 207-sample data set and used for maximum likelihood (ML) tree building (Fig. 2A). The SNV-based ML phylogeny revealed 6 distinct evolutionary clusters, which were broadly congruent with RT/ST lineage and toxin gene profile: (i) a large group of 99 strains primarily comprising RT126 and RT078 (designated the RT126/078 cluster), (ii) a group of 33 strains comprising exclusively RT033 and RT288 (the RT033/288 cluster), (iii to v) three distinct groups of strains (44, 3, and 21, respectively), belonging predominantly to RT127 (RT127 clusters I to III), and (vi) a divergent group of 7 ST258 strains (the ST258 cluster). Individual phylogenies for each cluster, annotated with metadata, are presented in Data Set S1 at figshare.

In all clusters, there was a general absence of geographic grouping and significant overlap of clinical and nonclinical strains, a finding that supports similar RT- and MLST-based studies that have shaped the hypothesis that strains of ST11 common to humans, animals, and the environment share a recent evolutionary history (1, 3, 17). To provide ultrahigh resolution of this strain population and to examine this hypothesis further, the SNV phylogeny was investigated for signatures of clonal transmission. Following the standard approach of Eyre et al. (4, 18, 19), a species-specific molecular clock of 1 to 2 SNVs per genome per year was applied, with a cutoff of 0 to 2 core genome SNVs indicative of a plausible clonal transmission event (Fig. 2B). These thresholds have been shown to be congruent with cutoffs used for core genome MLST (cgMLST), which is based on 2,270 loci and uses a threshold of a difference of ≥7 alleles to define isolates as being unrelated, whereas a difference of ≤6 alleles is used to define isolates as likely to belong to the same clone (20).

Applying the threshold of Eyre et al., 25 clonal groups (CG1 to -25) were identified across the six phylogenetic clusters, defined as groups of two or more strains differing by ≤2 SNVs in their core genome (Table 1). These CGs comprised 25 distinct clones of major RTs 078, 126, 127, 033, and 288 and encompassed 117 isolates of clinical and nonclinical origins (Table 1). Overall, 19/25 CGs (76%) comprised strains isolated from the same host species, indicating intraspecies clonal transmission, while the remaining six CGs (24%) showed evidence of interspecies clonal transmission. Furthermore, many CGs revealed long-range transmission of C. difficile clones across local, national, and international distances (Table 1).

**TABLE 1:** Summary of intra- and interspecies clonal groups^*a*^

CG	Clone	*n*	Transmission	C. difficile source	Range	Yr	Comments
1	RT078	3	Intraspecies	CDI	International and national	2010–2012	3 HCFs in Rotterdam, Netherlands, and in Australia (WA and NSW)
2	RT078	2	Interspecies	Piglet feces and asymptomatic farm worker	Local	2011	1 farm in Heino, Netherlands; corroborates work of Knetsch et al. (4)
3	RT126	6	Intraspecies	CDI	International and national	2007–2013	3 HCFs in Australia (NSW, WA, and VIC) and 1 HCF in Tainan, Taiwan
4	RT126	12	Intraspecies	CDI	International and national	2006–2013	3 HCFs in Toscana region of Italy, 1 HCF in Illinois, and 3 HCFs in 2 Australian states (WA and NSW)
5	RT126	2	Intraspecies	CDI	Local	2012	1 HCF in NSW, Australia
6	RT126	2	Intraspecies	CDI	Local	2006	1 HCF in NSW, Australia
7	RT126	2	Intraspecies	CDI (both CA-CDI)	Local	2011	2 distinct HCFs in NSW and VIC, Australia
8^*b*^	RT078	2					QC^*c*^
9	RT078	2	Intraspecies	CDI (1 CA-CDI)	National	2013–2016	2 distinct HCFs in WA and NSW, Australia
10	RT078	2	Intraspecies	CDI	National	Unknown	1 HCF in Illinois
11	RT126	7	Interspecies	CDI, calf feces and calf carcass washing	National	2012–2013	3 distinct farms in 2 Australian states (VIC and QLD) and 1 HCF in NSW, Australia
12	RT127	24	Interspecies	CDI (one CA-CDI), calf feces and calf carcass washing	National	2011–2014	5 distinct farms in 2 Australian states (VIC and NSW) and 5 distinct HCFs in 2 Australian states (NSW and WA)
13	RT127	4	Intraspecies	CDI (1 pediatric)	National	2006–2008	3 distinct HCFs in Australia (NSW and WA)
14	RT127	3	Interspecies	CDI, calf feces	National	2011–2012	1 HCF in NSW, Australia, and 1 farm in QLD, Australia
15	RT127	2	Intraspecies	CDI (both CA-CDI)	Local	2006	1 HCF in VIC, Australia
16	RT127	6	Intraspecies	CDI	Local	2010	1 HCF (ward) in Tokyo, Japan
17	RT127	2	Intraspecies	CDI	Local	2011–2012	1 HCF in Tainan, Taiwan
18	RT127	4	Intraspecies	CDI	Local	2005–2006	3 distinct HCFs in WA, Australia
19	RT126^*b*^	5	Interspecies	CDI (1 pediatric, 1 CA-CDI) and kangaroo feces	Local	2009–2012	3 distinct HCFs and 1 veterinary hospital, all in WA, Australia (26)
20	RT033	3	Intraspecies	CDI	Local	2006	1 HCF in VIC, Australia
21	RT033	2	Intraspecies	CDI	Local	1980–1982	1 HCF in VIC, Australia
22	RT033/288	6	Interspecies	CDI (1 pediatric), calf feces, and calf carcass washing	National	2012–2013	4 distinct farms in 3 Australian states (NSW, VIC, and QLD) and 2 HCFs in WA/VIC, Australia^*e*^
23	RT033	6	Intraspecies^*d*^	Piglet feces, soil irrigated with effluent, and treated liquid effluent	Local	2012–2015	2 farms in SA, Australia
24	RT033	6	Intraspecies	CDI	National	2011–2013	6 CDI epidemiologically unrelated cases from 6 distinct hospitals in the northern, central, and southern regions of France, previously described by Eckert et al. (25)
25	RT288	2	Intraspecies	Calf carcass washing	Local	2013	1 farm in SA, Australia

a CG, clonal group; RT, PCR ribotype; CDI, Clostridium difficile infection; CA-CDI, community-associated CDI; HCF, health care facility; SA, South Australia; QLD; Queensland; NSW, New South Wales; VIC, Victoria; WA, Western Australia; QC, quality control.

b ST258.

c M120 (accession no. NC_017174) was used as a reference chromosome for read mapping and SNV calling. For quality control purposes, the original paired-end reads for this strain (accession no. ERR027342) were obtained from the study by He et al. (41) and processed and analyzed as an additional test genome. SNV analysis correctly showed C137 and M120 to be indistinguishable (zero core genome SNV differences).

d Transmission between piglets (feces) and environment (soil/effluent).

e Notably, one strain is RT288, and the remainder are RT033.

### *C. difficile* ST11 possesses an extensive AMR repertoire.

**FIG 3:**
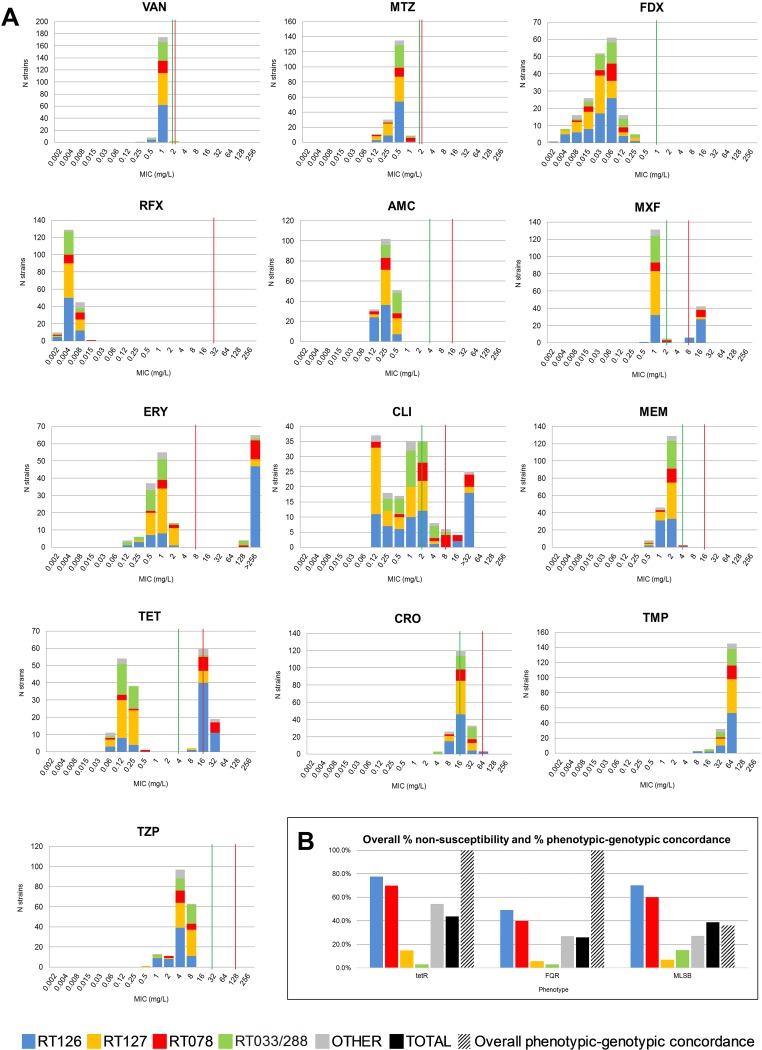
*In vitro* antimicrobial susceptibility. (A) MIC distributions for 13 antimicrobial agents against 185 C. difficile. VAN, vancomycin; MTZ, metronidazole; FDX, fidaxomicin; RFX, rifaximin; AMC, amoxicillin-clavulanate; CLI, clindamycin; ERY, erythromycin; CRO, ceftriaxone; MEM, meropenem; MXF, moxifloxacin; TET, tetracycline; TZP, piperacillin-tazobactam; TMP, trimethoprim. Where available, established susceptible and resistant breakpoints are indicated by vertical green and red lines, respectively. (B) Plots showing overall percentage of nonsusceptibility and percentage of phenotypic-genotypic concordance for the TetR (tetracycline-resistant), FQR (fluoroquinolone-resistant), and MLS_B_ (macrolide-lincosamide-streptogramin B-resistant) phenotypes.

The 207 C. difficile genomes were screened *in silico* for acquired and intrinsic resistance determinants. Of these strains, 185 were available for *in vitro* phenotypic testing. Summary MIC distributions for 13 antimicrobial agents, by RT lineage, are presented in Fig. 3A, and the MIC range, MIC_50_, MIC_90_, and geometric mean (GM) for all RT lineages are presented in Data Set S1 at figshare. All strains including ST258 were fully susceptible to vancomycin, metronidazole, fidaxomicin, rifaximin, amoxicillin-clavulanate, trimethoprim, and piperacillin-tazobactam (Fig. 3A; see Data Set S1 at figshare).

**FIG 4:**
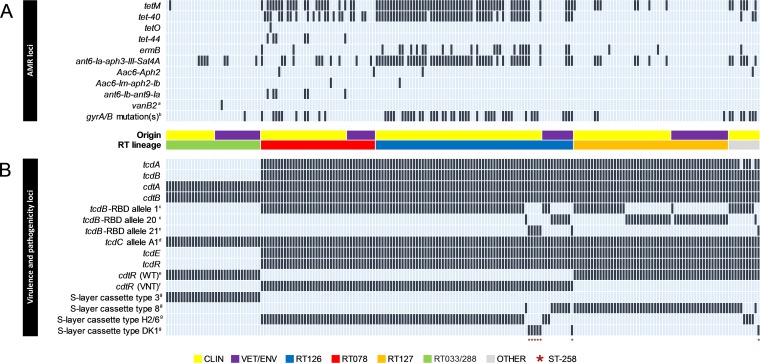
Comparative analysis of antimicrobial resistance (AMR) and virulence loci. Shown are heat maps visualizing the distribution of AMR (A) and virulence/pathogenicity (B) loci across the 207-genome data set. Presence is indicated by black bars and absence by light blue bars. Strains are arranged from left to right according to RT lineage: RT033/288 (green; *n = *33), RT078 (red; *n = *40), RT126 (blue; *n = *69), RT127 (orange; *n = *54), and other (gray; *n = *11). Strain origin is also indicated in yellow (clinical) and purple (veterinary/environmental). Superscript a indicates results comprising syntenic *vanXB*, *vanB*, *vanHB*, *vanW*, *vanYB*, *vanSB*, and *vanRB* genes. Superscript b indicates the combination of QRDR mutations in *gyrA* (Thr82Ile) and *gyrB* (Ser366Val, Ser416la, Asp426Asn, and Glu466Val). Superscript c indicates results are according to the scheme of Dingle et al. (21). (Alleles 20 and 21 are novel and were identified in this study.) Superscript d indicates results are according to the scheme of Curry et al. (23) (characterized by C→T substitution at nucleotide 184 and an in-frame deletion of 39 bp at nucleotide positions 341 to 379). Superscript e indicates the wild-type (WT) 747-bp *cdtR* allele. Superscript f indicates the variant (VNT) 324-bp *cdtR* allele. Superscript g indicates results are according to the scheme of Dingle et al. (21) (characterized by diversity in *slpA*, *cwp66*, *cd2790*, *cwp2*, and *secA2*). (Cassette type DK1 is novel and was identified in this study but has yet to be assigned an official number by the curators of the Bacterial Isolate Genome Sequence Database.)

Overall, 48.1% of strains showed phenotypic resistance to one or more of the agents tetracycline, moxifloxacin, erythromycin, and clindamycin, 25.4% of which (predominantly RT126/078), were multidrug resistant (MDR): i.e., resistant to ≥3 of these agents. Resistance was conferred by a diverse selection of acquired AMR genes (479 individual genes of 22 types across 4 antimicrobial classes) and intrinsic mutations in DNA gyrase subunit genes (Fig. 4A; see Data Set S1 at figshare). The distribution of AMR genotypes and the key genetic features of major AMR-encoding transposons found in this population are presented in Fig. 4A and Table 2, respectively.

**TABLE 2:** Major AMR-encoding transposons^*a*^

Tn (accession no.)	AMR locus and mechanism	Key genetic features and architecture	Reference
Tn*6194* (HG475346)^*b*^	*ermB*: methylation of 23S rRNA of bacterial 50S ribosomal subunit, thereby reducing binding affinity of MLS_B_ class antimicrobials	28,014 bp in size and 35 CDSs	44
		1 copy of *ermB*, distinguishing it from Tn*5398*, which has 2 copies^*c*^	
		Recombination module comprising *int* (1,446 bp) and *xis* (258 bp) genes	
		*int*/*xis* genes invariably found adjacent to a tRNA gene (Arg)	
		Contains toxin (ζ [159 bp]), antitoxin (ε [273 bp]), and 3′ cell surface protein (3,045 bp) genes	

Tn*6190* (FN665653)	*tetM*: mimics ribosomal elongation factors, protecting against antitranslational activity of tetracyclines	18,032 bp in size^*d*^	41
		Contains a recombination module comprising *int* (1,218 bp) and *xis* (204 bp) genes, distinguishing it from Tn*5397*, which contains a site-specific recombinase gene, *tndX*^*e*^	
		*orf12* (encoding a leader peptide) is absent and lacks the 1,831-bp group II intron in *orf14*, both characteristic features of Tn*5397*	

Tn*6164* (FN665653)^*f*^	*tet-44*: mimics ribosomal elongation factors, protecting them from antitranslational activity of tetracyclines	106,711 bp, containing 5 distinct modules (A to E) and 90 ORFs originating from diverse bacterial genera (average GC content of 34%)	14
		Module A (7.3 kb), located at the 5′ end of the element, contains a restriction-modification system.	
		Module B (39.5 kb) contains a complete prophage of *Thermoanaerobacter* sp. strain 513X (CP002210).	
		Module C (4.5 kb) contains part of plasmid originating from E. faecalis (pEF418 [AF408195.1]) including spectinomycin adenyltransferase gene *ant9-Ia*.	
		Module D (4.5 kb) is located entirely within module E, shows homology to a pathogenicity island of Campylobacter fetus, and harbors *tet-44* and *ant6-Ib* genes.	
		Module E (51 kb) is located at the 3′ end of the element and homologous to an entire conjugative transposon from Streptococcus pneumoniae, Tn*1806*.	

Tn*1549* (KU558763)	*vanB2* operon (*vanXB*, *vanB*, *vanHB*, *vanW*, *vanYB*, *vanSB*, and *vanRB*): biosynthesis of modified peptidoglycan precursors (e.g., d-Ala-d-Lac or d-Ala-d-Ser) to which vancomycin shows reduced binding	42,375 bp in size and 38 ORFs with homology and synteny with Tn*1549* (AF129329)^*g*^	22
		Recombination module comprising int (1,194 bp) and xis (201 bp) genes	
		The central AMR domain comprises the *vanB2* operon with syntenic *vanXB*, *vanB*, *vanHB*, *vanW*, *vanYB*, *vanSB*, and *vanRB* genes.	
		However, the *vanRB* genes (encoding a cytoplasmic response regulator) are interrupted by loci from Bacillus megaterium (Bm3R1 [582 bp]) and Bacillus cereus (*orf7* [1,032 bp]), respectively.	
		The aberrant *vanRB* genes are likely responsible for the cryptic phenotype observed (22).	
		Defining the left (L) and right (R) terminal ends of the element were 11-bp inverted repeats matching those found in Tn*1549* and likely representing excision/integration sites.	

a Tn, transposon; MLS_B_, macrolide-lincosamide-streptogramin B; *int*, integrase; *xis*, excisionase. ORF, open reading frame.

b Previously termed CTnCD3a (41).

c Tn*5398,* the predominant *ermB*-containing element in C. difficile (40).

d Size based on reported homology to Tn*916* (14).

e Tn*5397,* the predominant *tetM*-containing element in C. difficile (40).

f Position in C. difficile M120 genome (418525 to 525236).

g Tn*1549*, conjugative transposon linked with the emergence and global dissemination of vancomycin-resistant enterococci (22).

### (i) Tetracycline resistance.

Nonsusceptibility to tetracycline was seen in 43.7% (*n = *81/185 [Fig. 3B]) of strains and varied widely with RT lineage (RT126, 77.6%; RT078, 70.0%; other, 54.5%; RT127, 14.9%; and RT033/288, 3.0%; *P* < 0.0001 [see Data Set S1 at figshare]). All ST258 strains were susceptible to tetracycline. One or more tetracycline resistance genes (*tetM*, *tet-40*, *tetO*, and *tet-44*) were identified in 47.8% (*n = *99/207) of sequenced genomes, and the tetracycline genotype showed concordance with the tetracycline phenotype in all 185 strains tested (Fig. 3B and 4A; see Data Set S1 at figshare). All *tetM*-positive strains harbored Tn*6190*, a conjugative element closely related to Tn*916* from Enterococcus faecalis (Table 2). For *tet-40*, no discernible transposons were identified; however, all 75 *tet-40* genes in this population were conserved and shared 100% sequence identity (seqID) and flanking regions with *tet-40* sequences from the rumen species Megasphaera elsdenii (CP009240) and Streptococcus suis (KC790465.1). The single *tet-O* gene found in an RT078 strain shared 100% and 99% seqID with *tet-O* from Campylobacter jejuni and S. suis (CP012911.1). Six RT078 strains carried *tet-44* on a conjugative transposon, Tn*6164* (Table 2).

### (ii) Fluoroquinolone resistance.

Nonsusceptibility to moxifloxacin was 25.9% (*n = *48/185 [Fig. 3B]) and varied widely with RT lineage (RT126, 49.3%; RT078, 40.0%; other, 27.0%; RT127, 5.6%; and RT033/288, 3.0%; *P* < 0.0001 [see Data Set S1 at figshare]). All ST258 strains were susceptible to moxifloxacin. Full-length sequences for *gyrA*/*B* were characterized for polymorphisms within their quinolone resistance-determining regions (QRDRs) using the scheme of Dingle et al. (21). Two alleles were identified for *gyrA* (*gyrA-2* and -*31*) and four for *gyrB* (*gyrB-12*, -*39*, -*93*, and -*96*). Of these, *gyrA*-*31* and *gyrB*-*39*, -*93*, and -*96* had mutations leading to nonsynonymous amino acid changes within the *gyrA*/*gyrB* QRDRs resulting in fluoroquinolone resistance (FQR; 100% phenotype-genotype concordance [Fig. 3B and 4A]).

### (iii) MLS_B_ resistance.

Overall nonsusceptibility to clindamycin and/or erythromycin was 38.9% (*n = *72/185 [Fig. 3B]). Nonsusceptibility to these agents varied widely with RT lineage (clindamycin, RT078, 55.0%; RT126, 31.3%; other, 27.3%; RT033/288, 15.2%; and RT127, 5.6%; *P* < 0.0001; erythromycin, RT126, 70.2%; RT078, 60.0%; other, 18.0%; RT033/288, 11.3%; and RT127, 7.0%; *P* < 0.0001 [see Data Set S1 at figshare]). All ST258 strains were susceptible to clindamycin and/or erythromycin. The 23S rRNA methyltransferase gene, *ermB*, was found in 13.0% of strains (*n = *27/207 [Fig. 4A; Data Set S1]). All *ermB*^+^ strains were negative for Tn*6215*, Tn*6218*, and the most common *ermB*-carrying element in this species, Tn*5398* (1). Of the 27 *ermB^+^* strains, 77.8% (*n = *21) harbored conjugative transposon Tn*6194* (HG475346.1). Of the six remaining strains, four showed significant homology (99% seqID) to a 12-kbp AMR gene cluster from Campylobacter coli (KT953380.1). Overall, *in silico* AMR genotyping was a poor predictor of AMR phenotype, with only 36.1% of MLS_B_^+^ (macrolide-lincosamide-streptagramin B-positive) isolates harboring *ermB* (Fig. 3B). The remainder were also negative for *ermA* and *ermC*, ribosomal proteins (L4/L22), and 23S rRNA gene mutations, suggesting an alternative AMR mechanism (data not shown).

### (iv) Aminoglycoside resistance.

One or more aminoglycoside/streptothricin resistance genes were found in 45.4% (*n = *94) of isolates (Fig. 4A; see Data Set S1 at figshare). The *aph3-III–sat4A–ant6-Ia* cassette was present in 39.6% (*n = *82) of isolates (Fig. 4A) and shared 99% seqID to a 7,269-bp fragment of an MDR cassette from Erysipelothrix rhusiopathiae (KP339868.1). The genetic context for *ant6-Ib* and *ant9-Ia* (present only in six strains of RT078) was Tn*6164*, the same 106-kb genetic island harboring *tet-44* (14) (Fig. 4A and Table 2).

### (v) Other resistance loci.

All isolates contained the β-lactamase-inducing penicillin-binding protein gene *blaR* (CD630_04700), the efflux resistance gene *cme* (CD630_31980), and in a single isolate, the lincomycin resistance gene *lnuC* (AY928180.1). No *rpoB* mutations were detected, corroborating the rifaximin phenotype. Also, none of the 207 ST11/258 genomes harbored a *vanGCD* operon (1). However, as we have previously described (22), a single RT033 strain isolated from an Australian veal calf at slaughter harbored syntenic vancomycin resistance genes *vanXB*, *vanB*, *vanHB*, *vanW*, *vanYB*, *vanSB*, and *vanRB* (Fig. 4A and Table 2), but did not show any reduced susceptibility to vancomycin *in vitro* (MIC, 1 mg/liter), possibly a result of an aberrant *vanRB* operon. This “vanB2-like” operon was carried on an ∼42-kb Tn*1549*, a conjugative transposon linked with the emergence and global dissemination of vancomycin-resistant enterococci, the first such finding in C. difficile (22).

### Clade 5 sublineages show significant diversity in key virulence loci.

*In silico* genotyping confirmed that the pathogenicity locus (PaLoc) genes *tcdA*, *tcdB*, *tcdE*, and *tcdR* were present in all large clostridial toxin (LCT)^+^ RT lineages, but absent from LCT^−^ RTs 033 and 288 (Fig. 4B). The *tcdC* gene was conserved across all strains and contained genetic changes that result in an aberrant, significantly truncated TcdC: a C→T substitution at nucleotide 184 and an in-frame deletion of 39 bp at nucleotides 341 to 379 (23).

**FIG 5:**
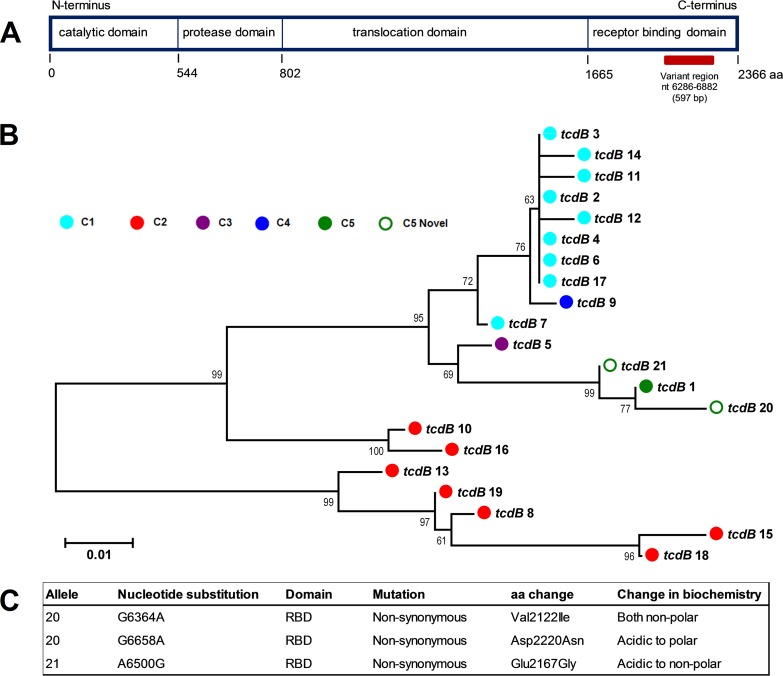
*tcdB* receptor binding domain (RBD) diversity. (A) Organization of the four functional domains of the 2,366-amino-acid TcdB protein. The 597-bp variable region within the C-terminus receptor binding domain (RBD) is indicated (horizontal red bar). The figure was adapted from Dingle et al. (21). (B) Neighbor-joining phylogeny for 21 currently described *tcdB* RBD alleles. Tips are colored according to the MLST clade (see the color key). Novel alleles identified in this study (open green circles) are clustered with known MLST clade 5 allele type 1 (reported to date in the RT078 and RT126 lineages). Each sequence is 198 amino acid residues in length. Sequences were aligned using MUSCLE, and the tree was generated in MEGA6 with evolutionary distances calculated using the Tajima-Nei model. The scale bar shows the number of amino acid substitutions per site. The tree is midpoint rooted and supported by 500 bootstrap replicates. (C) Summary of nucleotide and amino acid changes in novel *tcdB* RBD variants.

Genetic diversity in the C-terminus receptor binding domain (RBD) of *tcdB* was found with 70.6% of *tcdB^+^* isolates (*n = *144/174) harboring *tcdB* RBD allele type 1 (21) and the remainder carrying novel allele types 20 and 21, the latter exclusive to ST258 (Fig. 4B). These novel alleles share a recent evolutionary history with type 1 (Fig. 5B) and contain nonsynonymous substitutions that alter the amino acid sequence and biochemistry (Fig. 5C).

All strains harbored wild-type *cdtA*/*B* genes, but two variants of *cdtR* were identified: (i) a 324-bp *cdtR* allele was found exclusively in RTs 078/126, which, due to a stop codon at position 322, results in a truncated CdtR (from 248 to 108 amino acids [aa]) and (ii) a wild-type 747-bp *cdtR* allele was found only in non-078/126 strains (see Data Set S1 at figshare). Finally, characterized by diversity in *slpA*, *cwp66*, *cd2790*, *cwp2*, and *secA2* (21), 4 distinct S-layer cassettes were identified (including one novel type) that were broadly congruent with the RT and/or ST lineage (Fig. 4B).

### Clade 5 possesses a massive open pan-genome and a diverse population of temperate prophages.

**FIG 6:**
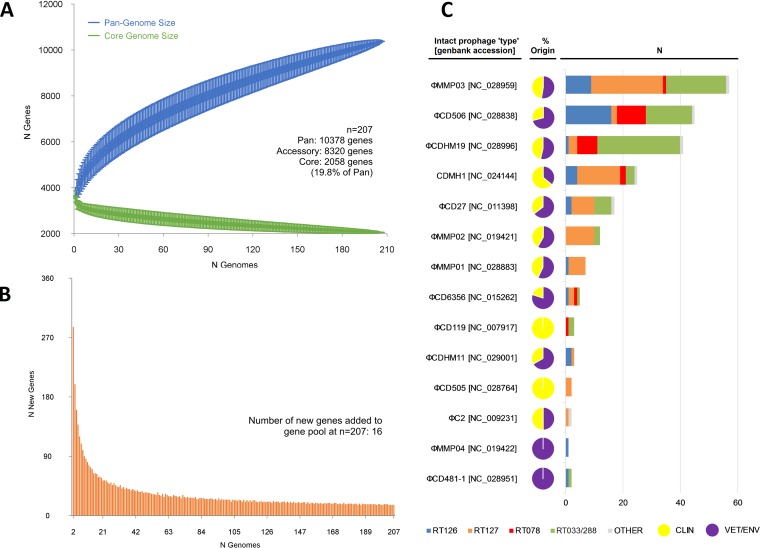
Pan-genome and prophage content. The total numbers of genes in the pan-genome (A) and core genome (B) are plotted as a function of the number of genomes sequentially added (*n = *207). (A) The pan-genome size is calculated at 10,378 genes at *n = *207 and displays characteristics of an open genome: (i) the trajectory of the pan-genome increases unboundedly as the number of genomes are added, and (ii) *B*_pan_ (≈γ [55]) was estimated as 0.46 (curve fit, *r*^2^ = 0.999). Box plots indicate the 25th and 75th percentiles, with medians shown as horizontal lines and whiskers set at the 10th and 90th percentiles. (B) Consistent with an open pan-genome, the core genome curve (*r*^2^ = 0.985) converges to 2,058 genes at *n = *207, where an average of 16 new strain-specific genes are contributed to the gene pool. Overall, the core genome accounts for just 19.8% of the total gene repertoire. (C) Summary of intact prophage content found in 207 C. difficile strains of ST11 and ST258. More prophages were found in LCT^−^ RTs versus LCT^+^ RTs, the RT127 lineage versus the RT126 and -078 lineages, and veterinary versus clinical strains (*P* < 0.001).

To quantify the entire genomic repertoire of the strain population, estimates of the pan-genome, core genome, and accessory genome were generated. The pan-genome was vast, comprising 10,378 genes, while the core and accessory genomes were 2,058 and 8,320 genes, respectively (Fig. 6A). The pan-genome showed characteristics of an “open” pan-genome (24). First, the pan-genome increased in size unboundedly with sampling of new genomes. At *n = *207, the pan-genome exceeded more than double the average number of genes found in a single ST11/258 genome (3,640) and the plot was yet to reach a plateau, indicating more sequenced strains are needed to capture the complete gene complement. Second, the number of new genes did not converge to zero upon sequencing of new strains (at *n = *207, an average of 16 new genes were contributed to the gene pool [Fig. 6A]). Finally, curve analysis using a power law regression model (24) showed the pan-genome was open (*B*_pan_ = 0.46 [Fig. 6A]). The core genome curve depicts a trend of core genome size contraction with progressive addition of sequential genomes (Fig. 6B), ultimately converging at 2,058 genes at *n = *207. Notably, the core genome accounted for just 19.8% of the total gene repertoire and 56.5% of an average ST11/258 genome (range, 49.8 to 60.2).

A total of 221 intact, 73 questionable, and 478 incomplete prophages were identified in the study population. A summary of the distribution and genetic features of intact prophages is shown in Fig. 6C and Data Set S1 (at figshare), respectively. The intact prophages comprised 14 “phage types,” ranging between 14.3 and 184.6 kb in length, with an average GC content of 29.9%, comparable to the average GC content for the C. difficile host (28.7%). The most common prophage was φMMP03 (*n = *57), followed by φCD506 (*n = *45), φCDHM19 (*n = *41), CDMH1 (*n = *25), φCD27 (*n = *17), φMMP02 (*n = *12), and φMMP01 (*n = *7) (Fig. 6C; see Data Set S1).

## DISCUSSION

C. difficile RT078 is a prominent ST11 sublineage that has established significant reservoirs in production animals worldwide (1, 2). It has also been recovered from various retail meat products in North America and Europe (2) and is a major cause of hospital-acquired (HA)- and CA-CDI in those regions (6, 8, 9). In many countries, although often to a lesser extent, ST11 RTs 126, 127, 033, and 288 have been recovered from humans with CDI (8, 10, 25–31), as well as livestock, slaughterhouses, and retail meat products (11–13, 30, 32–35).

Using large-scale high-resolution WGS, we provide novel insights into the evolution and genetic repertoire of ST11 and its close relative ST258. The global population structure largely mirrored the RT sublineage, with 6 discrete evolutionary clusters comprising highly genetically related strains unconstrained by geographic, temporal, or host species origin. Core genome analysis revealed intra- and interspecies clonal transmission of C. difficile in all the major ST11 sublineages and within the closely related novel clade 5 lineage ST258, which is potentially associated with CA-CDI and patients with hematological/oncological malignancies (26). Clones were spread across geographically distinct health care facilities and farms and indicated reciprocal long-range dissemination and possible zoonotic/anthroponotic transmission locally, nationally, and internationally. Our work supports and extends the findings of Knetsch and colleagues, who, not surprisingly, showed transmission of RT078 between a pig and pig farmer within the confines of a pig-rearing facility (4). In reconstructing the global RT078 population structure, they later revealed an intercontinental transmission network between humans and production animals of RT078 (15).

Our analysis also provided some interesting insights into the overall evolution of ST11/258. First, RTs 126 and 078 did not cluster into distinct subpopulations, suggesting they have coevolved, at least over their core genome, as a single heterogeneous lineage, a finding that supports their frequent reporting as a single RT group (1, 36). A similar observation was made for the LCT^−^ RTs 033 and 288, but the position of RT078/126 at the base of the phylogeny suggests they may be the more ancient of the clade 5 sublineages. Taken together, these phylogenetic analyses reveal a globally disseminated network of clones with the capability and proclivity for reciprocal clonal transmission between production animals and humans with CDI. Moreover, these findings challenge the existing paradigm and long-held conception that CDI is primarily a health-care-associated infection and provide compelling evidence that CDI is a zoonosis. While some human infections in Australia are likely a result of international travel (e.g., clones of RT078), our analyses also indicate a persistent community reservoir with extensive long-range domestic dissemination. Due to the high prevalence of C. difficile in neonatal cattle and pigs, the consumption of contaminated retail meats is a conceivable mechanism for transmission (2, 11). However, evidence from studying RT014, the most common RT found in humans and pigs in Australia, suggests a zoonotic transmission chain extending from the farrowing shed to the community (5, 12, 37). C. difficile can be found in 67% of Australian piglets (12), on 20% of retail root vegetables grown in soil containing animal feces (38), in 59% of public lawns in Western Australia (39), and in 30% of retail compost and manure (unpublished data), with RT014 comprising between 7 and 67% of isolates in these settings. In a manner analogous to human infection, excessive exposure to antimicrobials, particularly to cephalosporins, is driving the expansion of C. difficile in livestock populations worldwide and resulting in spillover of C. difficile into the environment and CDI in the community (37).

AMR can evolve rapidly in C. difficile and is a key factor driving genetic diversity and epidemiological changes in CDI (1). The ST11 lineage has a substantial AMR repertoire, characterized by high levels of phenotypic resistance to tetracycline, moxifloxacin, clindamycin, or erythromycin, predominantly within the RT 126/078 lineages.

TetR strains of C. difficile comprise up to 41% of European clinical isolates (40). We found 30% of ST11 isolates had a TetR phenotype conferred by efflux and ribosomal protective proteins, expressed by Tn*6190* (*tetM^+^*) and Tn*6164* (*tet-44^+^*). Tn*6190* has a strong affiliation with the RT126/078 lineages, present in 79.7% and 55.0% of strains in this study, respectively, and to date only reported in these RTs (14, 16, 41). Similarly, Tn*6164* has been found in RT078 only and, prior to this study, only within Europe. Corver et al. (14) suggest there may be an association between the presence of this genetic island and enhanced virulence in RT078 strains: CDI-associated mortality was more common in patients infected with C. difficile strains harboring Tn*6164* (29% versus 3%) (14). The association of these elements with RTs 078 and 126 could provide a fitness advantage, other than AMR, and be a contributing factor in their success compared to the less widespread LCT^−^ and RT127 lineages. Indeed, a recent study by Dingle et al. (57) provides compelling evidence that tetracycline selection played a crucial role in the rapid and recent international spread of RT078 clones.

The prevalence of FQR in European C. difficile populations can be as high as 40%, mainly associated with hospital outbreaks of RT027 strains (10, 42, 43). As with RT027, FQR might also be an important driver of clonal expansion in ST11. In our study, FQR was largely restricted to RT078/126 strains of human clinical origin and was notably absent in C. difficile from Australian livestock, reflecting the current restrictions on fluoroquinolone usage in food animals in this country (10).

WGS permits rapid prediction of an antimicrobial phenotype. We found concordance between MICs and *in silico* AMR screening was high for the tetracycline and fluoroquinolone phenotype (100%), but poor for the MLS_B_ phenotype (36.1%). Over a third of all strains, again principally RTs 126/078, had an MLS_B_ phenotype, yet only 36% of these harbored *ermB^+^* Tn*6194*, the first such report from humans in Australia or from animals elsewhere in the world. This element is the most common *ermB*-containing element in European human clinical isolates, has interspecies transfer proficiency, and is one of the defining genetic features of epidemic RT027 (1, 40, 42). The explanation for the MLS_B_^+^ *ermB* mutant is not clear. Such strains did not contain alterations in ribosomal proteins or 23S rRNA, both alternative mechanisms from which reduced susceptibility to macrolides and lincosamides can arise (43). Previously, Spigaglia et al. (43) showed that treatment of MLS_B_^+^ *ermB* mutant strains with two efflux pump inhibitors did not lead to reductions in MICs. Therefore, it appears that in the ST11 lineage *ermB* is not the primary mechanism underlying the MLS_B_ phenotype, and other mechanisms, potentially efflux but possibly novel, may be at play.

Many of the underlying AMR elements we identified show provenance in different commensal species residing within the gut of pigs and cows. Some of these elements are fully capable of both intraspecies transfer to different C. difficile RTs and interspecies transfer to other genera (41, 42, 44). Taken together with our microevolutionary analysis, this suggests that ST11 and perhaps also ST258 has the capability and propensity to move between production animals and humans and in doing so can possibly access and exchange DNA with an enormously diverse metagenome found in the human and pig (monogastric) and cow (ruminant) gut microbiota. The high prevalence of cryptic aminoglycoside and streptothricin resistance gene clusters originating from *E. rhusiopathiae* is intriguing as C. difficile is inherently resistant to aminoglycosides. It most likely reflects a long history of reciprocal lateral gene transfer within the monogastric and ruminant gut environments, with C. difficile likely serving as a reservoir of AMR loci, for both other C. difficile lineages and other commensal genera.

Analysis of genes common to the PaLoc, CdtLoc, and S-layer identified several new alleles and many instances of RT/ST lineage-specific diversity. These findings may indicate evolution within different host environments and possibly explain differences in virulence potential between more (078 and 126) and less (033, 288, and 127) successful lineages. For example, CDT^+^ strains are associated with more severe diarrhea, a higher case-fatality rate, and refractory disease (45). The high sequence diversity in *cdtB* (encoding the binding component of CDT), particularly between the LCT^+^ CDT^+^ and LCT^−^ CDT^+^ lineages, may reflect differences in host cell binding *in vivo* for these different genotypes. Furthermore, DNA binding for the response regulator CdtR is predicted to occur within the C-terminal domain (45). It is possible that the truncated CdtR found exclusively in RTs 078 and 126 may be nonfunctional as a positive regulator of *cdtA*/*B*, conferring a fitness advantage in these more virulent/successful lineages. Similarly, one of the novel *tcdB* RBD variants identified in this study (allele 21) was unique to ST258. The RBD of TcdB is a critical region for interaction with host epithelial cell membranes, and variations within this region have been associated with enhanced virulence (1, 21). The significant changes in amino acid biochemistry in this region could result in an alternate, potentially less virulent (less successful), disease phenotype compared to the more globally disseminated ST11. Finally, the S-layer plays a central role in adaption to life in the gastrointestinal tract and evolves in response to host immunological selection (21). The four distinct S-layer cassettes identified were highly congruent with the RT and/or ST lineage, possibly reflecting evolution in different (original) host species. Moreover, S-layer cassette typing could be a useful additional discriminatory typing tool for the numerous RTs within ST11 and clade 5.

Comprising just 19.8% (2,058 genes) of its genetic repertoire, the core genome of C. difficile ST11/258 is remarkably small, a finding that supports earlier studies describing ultralow genome conservation in this species (16 to 40%) (1). These values are considerably lower than those for other pathogens known to have significant genomic variability: e.g., Helicobacter pylori (∼59%), Campylobacter jejuni (∼53%), Streptococcus pneumoniae (∼47%), Escherichia coli (∼40%), and Legionella pneumophila (∼33%) (1). At almost 10,400 genes, the pan-genome is comparable with that of Salmonella enterica (10,000 genes), one of the most diverse species in the bacterial kingdom (46). Underlying the incredible diversity seen in the accessory genome is a substantial population of *Siphoviridae* and *Myoviridae*, including φC2, φCD38-2, φCD27, φMMP02, φCDHM1, φMMP03, φCD506, and φCDHM19. These temperate tailed prophages share a similar GC to their host (28 to 30%) and have coevolved with C. difficile over very long periods (1). Studies have shown that in their lysogenic form, these phages are able to influence the expression of multiple genes associated with the fitness and virulence of the host bacterium during infection, including modulation of quorum-sensing, flagellar assembly, AMR transduction, and toxin production (1, 47).

There are limitations to this work. While widely recognized as the current standard approach for studies of pathogen transmission (18, 19, 48, 49), the molecular clock for any species is an approximation based on within-host variation and the assumption of a constant rate of evolution. Therefore, it does not account for the genetically quiescent nature of C. difficile spores and may underestimate the evolutionary distance between strains (19). Also, with comparable resolution to SNV analysis and the added bonus of standardized nomenclature, cgMLST could be used as a comparator typing tool in future studies (20). Last, we acknowledge that plasmids were not investigated. Differentiation of large plasmids and some prophage elements is difficult, but future studies that screen large cryptic plasmids such as pDLL3026 may provide further insights into the evolution of clade 5 strains and their prophages.

In summary, the One Health paradigm, connecting the health of humans to the health of animals and their shared environments, represents the optimal approach for understanding the epidemiology and evolution of C. difficile, as well as improving strategies to curtail the growing public health threat posed by CDI. Better communication and coordinated efforts between public health authorities, veterinary medicine, and agriculture will be key in developing interventions aimed at reducing the levels of C. difficile spores in the environment. These include curtailing the use of late-generation cephalosporins, immunization, environmental cleaning (e.g., sporicidal treatment of effluent) and discontinuing the practice of slaughtering neonatal calves (37, 50).

Our study demonstrates the zoonotic potential of ST11 and its close relative ST258 and provides a framework for future epidemiological and experimental studies of other livestock- or agricultural-associated lineages of C. difficile. Moreover, our findings challenge the long-held misconception that CDI is primarily nosocomial in origin and clearly emphasize the need for continued genetic and phenotypic surveillance of C. difficile from different ecological niches. As we have shown here, WGS provides the ultrafine-scale resolution needed to decipher cryptic CDI transmission pathways and identify emerging clones, as well as changes in AMR and key virulence loci.

## MATERIALS AND METHODS

### Bacterial isolates.

A detailed summary of isolates (*n = *185), genomes (*n = *207), and associated epidemiological data is provided in Data Set S1 at figshare (https://doi.org/10.6084/m9.figshare.4822255). Briefly, strains of human clinical origin (*n = *150) were sourced from patients with CDI in 62 unique health care facilities/hospitals in Australia (*n = *92), Europe (*n = *36), Asia (*n = *14), North America (*n = *7), and New Zealand (*n = *1) and collected between 1980 and 2016. C. difficile strains of veterinary and environmental origin (*n = *57) were sourced from 16 unique farms, abattoirs, veterinary laboratories, and one organic market in Australia (*n = *43) and Europe (*n = *14) between 2007 and 2015. C. difficile strains were selected based on their RT belonging to ST11 (e.g., RTs 033, 078, 126 and 127, and 288), as well as several novel ST11 RTs (prefixed with “QX”). RT and toxin gene profiles for all 185 available C. difficile isolates were reconfirmed by PCR assays as previously described (13) (Fig. 1).

### Whole-genome shotgun sequencing.

Genomic DNA was extracted from a 48-h blood agar subculture of C. difficile using a QuickGene DNA tissue kit (Kurabo Industries, Osaka, Japan). A total of 185 strains were subjected to WGS using the MiSeq and HiSeq platforms and standard Nextera XT libraries (Illumina, San Diego, CA) (5). For comparative analysis, the genomes of 22 previously sequenced human clinical ST11 strains from European studies (4, 36, 41) were also included in this study. Accession numbers for WGS data are provided in Data Set S1 at figshare.

### Microevolutionary analysis.

Core genome single nucleotide variant (SNV) analysis followed the “gold standard” approach of Eyre et al. (18), as recently described (5). Briefly, trimmed reads were mapped to the finished chromosome of C. difficile strain M120 (ST11 [accession no. NC_017174]) using Smalt v0.7.6 (www.sanger.ac.uk/resources/software/smalt/). Candidate SNVs were filtered for quality and coverage and called across all mapped sites using SAMtools v0.1.12-10, with subsequent removal of indels and masking of repetitive regions, mobile genetic elements, and recombination regions (5). This approach resulted in a final set of 1,076 concatenated SNVs in “clonal frame,” which was used (i) to calculate pairwise core genome SNV differences between isolates and (ii) to generate maximum likelihood phylogenies. Trees were produced using RAxML v8.1.23 (51) with a generalized time-reversible (GTR) model of evolution and CAT approximation of rate heterogeneity and curated using FigTree v1.4.2 (52) and iToL v4 (53).

### Comparative genomic analysis.

Sequence reads were interrogated for MLST and acquired AMR genes using the pubMLST and ARG-ANNOT databases, respectively, compiled within SRST2 v0.1.8 (54). Genome assembly and annotation and comparative analysis of transposons (Tns), prophages, and virulence loci were performed *in silico* as previously described (5). Annotated genomes were used as input for pan-genome analysis with Roary v3.6.0 and PanGP v1.0.1 as previously described (5). Definitions of the core and pan-genome and estimates of the irrespective size and trajectory were made using models and regression algorithms proposed by Tettelin and colleagues (55), as previously described (5).

### Antimicrobial susceptibility testing.

MICs for 13 antimicrobials were determined using the CLSI agar dilution methodology (56). Clinical breakpoints were applied as recommended by CLSI (for amoxicillin/clavulanate, ceftriaxone, clindamycin, clindamycin, erythromycin, meropenem, moxifloxacin, piperacillin-tazobactam, and tetracycline), EUCAST (for vancomycin and metronidazole), and the European Medical Agency (for fidaxomicin) as previously described (5). A MIC of ≥32 mg/liter was used to define resistance to rifaximin (5), and there are no published breakpoints for trimethoprim.

### Statistical analysis.

Where appropriate, statistical significance was determined using a χ^2^ test, *t* test, or Kruskal-Wallis *H* test, using a cutoff *P* value of ≤0.05.

### Data availability.

All supplemental data for this article (Data Set S1) and 207 annotated C. difficile genome assemblies are hosted at the online digital repository figshare, available at https://doi.org/10.6084/m9.figshare.4822255.
